# Ethanol Extract of *Hizikia fusiforme* Induces Apoptosis in B16F10 Mouse Melanoma Cells through ROS-Dependent Inhibition of the PI3K/Akt Signaling Pathway

**DOI:** 10.31557/APJCP.2020.21.5.1275

**Published:** 2020-05

**Authors:** Cheol Park, Hyesook Lee, Hyun Hwangbo, Seon Yeong Ji, Min Yeong Kim, So Young Kim, Su Hyun Hong, Gi-Young Kim, Yung Hyun Choi

**Affiliations:** 1 *Department of Molecular Biology, College of Natural Sciences, Dong-eui University, Busan 47340, Republic of Korea. *; 2 *Anti-Aging Research Center, Dong-eui University, Busan 47340, Republic of Korea. *; 3 *Department of Biochemistry, College of Korean Medicine, Dong-eui University, Busan 47227, Republic of Korea. *; 4 *Laboratory of Immunobiology, Department of Marine Life Sciences, Jeju National University, Jeju 63243, Republic of Korea. *

**Keywords:** Hizikia fusiforme, B16F10 cells, apoptosis, ROS, PI3K/Akt

## Abstract

**Background:**

Previous studies have reported that *Hizikia fusiforme*, an edible brown seaweed, has diverse health-promoting effects; however, evidence for its anti-cancer potential is still lacking. In this study, we examined the effect of ethanol extract of *H. fusiforme *(EHF) on the proliferation of B16F10 mouse melanoma cells.

**Methods:**

Analyses of cell viability and apoptosis were performed to study the actions of EHF on B16F10 cells. Cellular reactive oxygen species (ROS) and mitochondrial membrane potential (*ΔΨm*) were measured using a flow cytometer. Western blot analysis was carried out to measure apoptosis and phosphoinositide 3-kinase (PI3K)/Akt signaling related proteins.

**Results:**

EHF treatment significantly decreased B16F10 cell viability, which was associated with induction of apoptosis. EHF activated caspase-8 and caspase-9, which are involved in the initiation of extrinsic and intrinsic apoptosis pathways, respectively, and also increased caspase-3 activity, a typical effect caspase, subsequently leading to poly (ADP-ribose) polymerase cleavage. In addition, EHF destroyed the integrity of mitochondria and increased Bax/Bcl-2 ratio, which contributed to cytosolic release of cytochrome c. EHF further enhanced intracellular levels of ROS and the addition of N-acetyl cysteine (NAC), a ROS inhibitor, significantly diminished EHF-induced mitochondrial dysfunction and growth inhibition. Moreover, EHF inactivated the PI3K/Akt signaling pathway and LY294002, a PI3K/Akt inhibitor, increased the apoptosis-inducing effect of EHF. However, increased apoptosis and reduced cell viability by simultaneous treatment of EHF and LY294002 were significantly attenuated in the presence of NAC.

**Conclusion:**

These results indicate that EHF induces apoptosis through activation of extrinsic and intrinsic apoptotic pathways and ROS-dependent inactivation of PI3K/Akt signaling in B16F10 cells.

## Introduction

Skin cancer, especially melanoma, is a highly metastatic aggressive cancer, and metastatic melanoma is the leading cause of death worldwide (Davey et al., 2016; Singh et al., 2017) To date, surgery, chemotherapy and radiotherapy, and combination therapies of them are standard choices for melanoma treatment, but it is important to apply appropriate alternatives or complementary therapies because of the side effects of these therapies (Kakavand et al., 2016; Russo et al., 2018). Although an understanding of the precise mechanisms on onset and progression, and pathological features of melanoma for successful treatment strategies is required, recent interest in the induction of various types of programmed cell death for melanoma treatment has been growing (Broussard et al., 2018; Pourhanifeh et al., 2019). 

Among conventional cell death mechanisms, apoptosis is one of the most actively studied areas of cancer treatment, which is largely divided into mitochondria-mediated intrinsic and death receptor (DR)-mediated extrinsic pathways (Kiraz et al., 2016; Pfeffer and Singh, 2018). The intrinsic pathway begins with the release of pro-apoptotic proteins, such as cytochrome c, from the mitochondria to the cytoplasm with increased mitochondrial permeability and activation of caspase-9. On the other hand, the extrinsic pathway is characterized by the activation of caspase-8 by the formation of the death-inducing signaling complex through the binding of death ligand to the cell surface DR. Activated caspase-8 and -9 as initiator caspases activate downstream effector caspases, including caspase-3, which ultimately induce apoptosis through cleavage of cellular substrates. In addition, these pathways are strictly regulated by a group of proteins composed of anti- and pro-apoptotic proteins such as Bcl-2 protein family proteins (Birkinshaw and Czabotar, 2017; Edlich, 2018). Meanwhile, apoptosis is precisely regulated by a wide variety of cellular signaling pathways. Among them, the phosphoinositide 3-kinase (PI3K)/Akt signaling pathway is involved in the inhibition of apoptosis and the promotion of cell growth, thus playing a key role in the pathogenesis of various tumors (Zhang et al., 2007; Yea and Fruman, 2013; Karimian et al., 2019). Moreover, there is a growing interest in reactive oxygen species (ROS), which induce apoptosis of cancer cells through dysregulation of the PI3K/Akt signaling pathway (Mi et al., 2016; Song et al., 2017; Guo et al., 2018). Therefore, inhibiting the PI3K/Akt signaling pathway, while promoting the generation of ROS, can be an attractive approach to cancer treatment. 

Recently, many studies have shown that marine algae have received great interest as potential resources for cancer chemoprevention and chemotherapy (Correia-da-Silva et al., 2017; Martínez Andrade et al., 2018). Among them, *Hizikia fusiforme*, an edible brown seaweed distributed in the northwest Pacific, including Korea, China, and Japan, has been widely used as a functional food for hundreds of years. Several previous studies have reported that extracts of *H. fusiforme *and its phytochemical compounds possess potent bioactivities which include anti-inflammatory, anti-diabetic, antioxidative, osteoprotective and immunostimulatory effects (Jeong et al., 2015; Jeong et al., 2016; Oh et al., 2016; Park et al., 2017; Kang et al., 2018; Wang et al., 2018). Although several studies, including our preliminary results, have reported that extract of *H. fusiforme *has anti-cancer effects (Kim et al., 2009; Kim and Choi, 2010; Son et al., 2018), the underlying mechanisms are still net well known. Therefore, in this study, we investigated the effect of ethanol extract of *H. fusiforme *(EHF) on the induction of apoptosis and evaluated whether its effect was associated with the ROS generation and PI3K/Akt signaling pathway in B16F10 mouse melanoma cells.

## Materials and Methods

*Preparation of EHF*

The EHF used in this study was prepared as described previously (Kim et al., 2009). In brief, *H. fusiforme *powder (100 g) was refluxed with 1 L of 70% ethanol solution by sonication for 24 h. After filtration through a glass filter funnel, the extract was concentrated by rotary vacuum evaporator (Buchi Labortechnik, Flawil, Switzerland), lyophilized and then stored at -80°C. The freeze-dried powder of ethanol extract (EHF) was dissolved in dimethylsulfoxide (DMSO, Sigma-Aldrich Chemical Co., Louis, MO, USA) to a final concentration of 100 mg/ml. The final concentration of DMSO in the medium was kept less than 0.1%, which has been shown nonlethal to the cells. The stock solution was diluted with cell culture medium to the desired concentration, prior to use.

*Cell culture and cell viability assay*

B16F10 cells purchased from the American Type Culture Collection (Manassas, MD, USA) were cultured in Dulbecco’s modified Eagle’s medium (DMEM) supplemented with 10% fetal bovine serum (FBS), 100 U/mL penicillin, and 100 μg/mL streptomycin (all from WelGENE Inc., Daegu, Republic of Korea) at 37°C in 5% CO_2_ humidified incubator. The cell viability was assessed by 3-(4,5-dimethyl-2-thiazolyl)-2,5-diphenyltetrazolium bromide (MTT) assay, as previously described (Ittiudomrak et al., 2019). In brief, B16F10 cells were seeded onto 96-well plates at a density of 1×10^4 ^cells/well. After 24 h of incubation, the cells were treated with the desired concentrations of EHF for 24 and 48 h, and the cells were then incubated with 50 μg/mL MTT solution (Invitrogen, Waltham, MA, USA) for 2 h. Formazan crystals were dissolved in DMSO, and the absorbance was measured using a microplate reader (VERSA Max, Molecular Device Co., Sunnyvale, CA, USA) at 540 nm. The morphological changes of cells were visualized with a phase-contrast microscope (Carl Zeiss, Oberkochen, Germany).

*Detection of apoptotic morphological changes*

Apoptotic cells containing nuclear fragmentation and chromatin condensation in the nuclei were detected by 4′,6′-diamidino-2-phenylindole (DAPI) staining. After treatment with EHF for 48 h, the cells were collected, washed with phosphate-buffered saline (PBS), and fixed with 3.7% paraformaldehyde (Sigma-Aldrich Chemical Co.) for 10 min at room temperature. The fixed cells were washed with PBS and stained with 1 μg/mL DAPI solution (Sigma-Aldrich Chemical Co.) for 10 min under light-shielded conditions. The cells were washed with PBS, and the fluorescence intensity was observed using a fluorescence microscope (Carl Zeiss). 

*Determination of apoptosis by a flow cytometer*

Quantitative evaluation of apoptosis induction was determined with a flow cytometer using annexin V/propidium iodide (PI) double staining. Briefly, the cells were harvested, resuspended in the supplied binding buffer and then stained with fluorescein isothiocyanate (FITC)-conjugated annexin V and PI (Becton Dickinson, San Jose, CA, USA) at room temperature for 20 min in the dark, according to the manufacturer’s protocol. Cell fluorescence was detected with a flow cytometer (Becton Dickinson), and acquisition was performed using Cell Quest Pro software (Becton Dickinson). The annexin^-^/PI^-^ cell population was considered normal, while the annexin V-FITC^+^/PI^-^ and annexin^+^/PI^+^ cell populations were considered indicators of apoptotic cells, respectively.

*Protein extraction and Western blot analysis*

Total protein was extracted from the cells using the Bradford Protein assay kit (Bio-Rad Laboratories, Hercules, CA, USA) according to the manufacturer’s protocol. NE-PER nuclear and cytoplasmic extraction reagents (Thermo Fisher Scientific Inc., Waltham, Utah, USA) were used for the preparation of mitochondrial and cytosolic extracts of cells by the manufacturer’s recommended protocol. After quantification of protein concentration using the Bio-Rad protein assay kit (Bio-Rad Laboratories, Hercules, CA, USA), equal amounts of proteins were separated by denaturing sodium dodecyl sulfate (SDS)-polyacrylamide gel electrophoresis, and then transferred onto polyvinylidene difluoride (PVDF) membranes (Millipore, Bedford, MA, USA). The membranes were blocked with 5% skim milk in Tris-buffered saline containing 0.1% Triton X-100 (TBST) for 1 h at room temperature, and probed with primary antibodies (Table 1), which were obtained from Santa Cruz Biotechnology, Inc. (Santa Cruz, CA, USA), Cell Signaling Technology, Inc. (Danvers, MA, USA) and Bioworld Technology, Inc. (Bloomington, MN, USA), to react with the blotted membranes at 4°C overnight. After washing with TBST, the membranes were incubated with the appropriate horseradish peroxidase (HRP)-conjugated secondary antibodies (1:500; cat. no. sc2004, goat anti rabbit immunoglobulin (Ig) G HRP; sc2005, goat anti mouse IgG HRP; Santa Cruz Biotechnology, Inc.) for 2 h. The expression of protein was detected by an enhanced chemiluminescence (ECL) kit (GE Healthcare Life Sciences, Little Chalfont, UK), and visualized by Fusion FX Image system (Vilber Lourmat, Torcy, France). All results were obtained in three independent experiments.

*Caspase activity*

The activity of caspases was measured using caspase activity assay kits (R and D Systems, Minneapolis, MN, USA), according to the protocol of the manufacturer. In brief, the cells were harvested and lysed in the lysis buffer provided in the kit. The supernatants were collected and then incubated with the supplied reaction buffer containing dithiothreitol with or without substrates [Ile-Glu- Thr-Asp (IETD) for caspase-8; Leu-Glu-His-Asp (LEHD) for caspase-9 and Asp-Glu-Val-Asp (DEAD) for caspase-3] labeled with p-nitroaniline (pNA) at 37°C for 2 h in the dark. The optical density of the reaction mixture was determined by absorbance at 405 nm using a microplate reader.

*Measurement of mitochondrial membrane potential (MMP, Δψm)*

To measure MMP, 5,5‘,6,6’-tetrachloro-1,1’,3,3’-tetraethyl-imidacarbocyanine iodide (JC-1) staining was performed. After treatment with EHF, 10 μM JC-1 (Sigma-Aldrich Chemical Co.) was added to the cells for 30 min at 37°C. Subsequently, the cells were washed with PBS. The amounts of MMP were detected at 488/575 nm using a flow cytometer, by following the manufacturer’s protocol. 

*Intracellular ROS generation*

The production of ROS was measured using 2′,7′-dichlorofluorescein diacetate (DCF-DA) as described previously (Lee et al., 2019). After treatment with EHF in the presence or absence of a ROS scavenger N-acetyl-L-cysteine (NAC, Invitrogen) for defined periods, 10 μM DCF-DA (Sigma-Aldrich Chemical Co.) was added to the incubated cells in the dark for 20 min. For the assessment of ROS production by a flow cytometer, the cells were washed with PBS and immediately analyzed using a flow cytometer at 480 nm/520 nm. 

*Statistical analysis*

All experiments were performed at least three times. Data were analyzed using GraphPad Prism software (version 5.03; GraphPad Software, Inc., La Jolla, CA, USA), and expressed as the mean ± standard deviation (SD). Differences between groups were assessed using analysis of variance followed by ANOVA-Tukey’s post hoc test, and p<0.05 was considered to indicate a statistically significant difference. 

## Results

*Inhibition cell viability by EHF in B16F10 cells*

**Figure 1:**
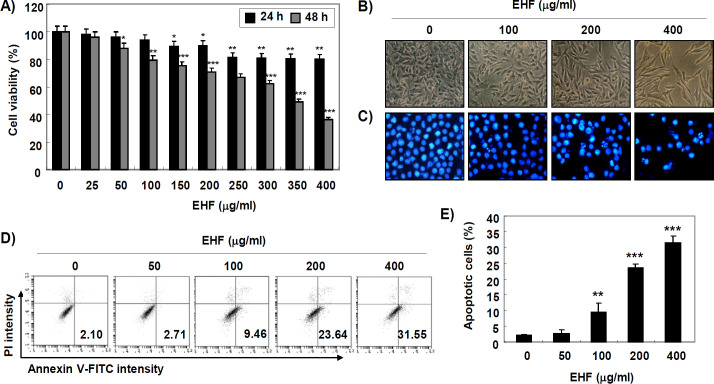
Inhibition of Cell Viability and Induction of Apoptosis by EHF in B16F10 cells. (A) After treatment of B16F10 cells with the desired concentration of EHF (0-400 μg/ml) for 24 and 48 h, cell viability was measured by MTT assay. Data are expressed as the mean ± SD (n=3). The statistical analyses were conducted using analysis of variance between groups (*P<0.05, **P<0.001 and ***P<0.0001 compared to control). (B-D) Cells were treated with various concentration of EHF (0-400 μg/ml) for 48 h. (B) Morphological changes of B16F10 cells were observed by a phase-contrast microscope. (C) After staining with DAPI solution, the stained nuclei were pictured under a fluorescence microscope. Representative photographs of the morphological changes are presented. (D) The cells were collected and stained with FITC-conjugated Annexin V and PI for flow cytometry analysis. (E) The percentage of apoptotic cells are shown as the mean ± SD (n=3). The statistical analyses were conducted using analysis of variance between groups (**P<0.001 and ***P<0.0001 compared to control).

In order to evaluate the cytotoxicity of EHF in B16F10 cells, the cells were incubated with different concentrations of EHF (0-400 μg/mL) for 24 and 48 h, and cell viability was assessed by an MTT assay. Figure 1A shows that EHF significantly reduced B16F10 cell viability in a time- and concentration-dependent manner. Under phase-contrast microscope, the phenotypic characteristics of EHF-treated cells showed irregular cell outlines, decrease of cell density, and increase of detached cells (Figure 1B). 

*Induction of apoptosis by EHF in B16F10 cells*

We determined whether the growth inhibition of B16F10 cells by EHF was associated with apoptosis induction. As shown in Figure 1C, EHF-treated cells displayed typical apoptotic nuclei, including chromatin condensation and revealing nuclear fragmentation. In addition, the results of annexin V/PI double staining also showed that EHF significantly increased the frequency of apoptotic cells in T24 cells (Figures 1D and E).

*Activation of caspases by EHF in B16F10 cells*

**Figure 2.**
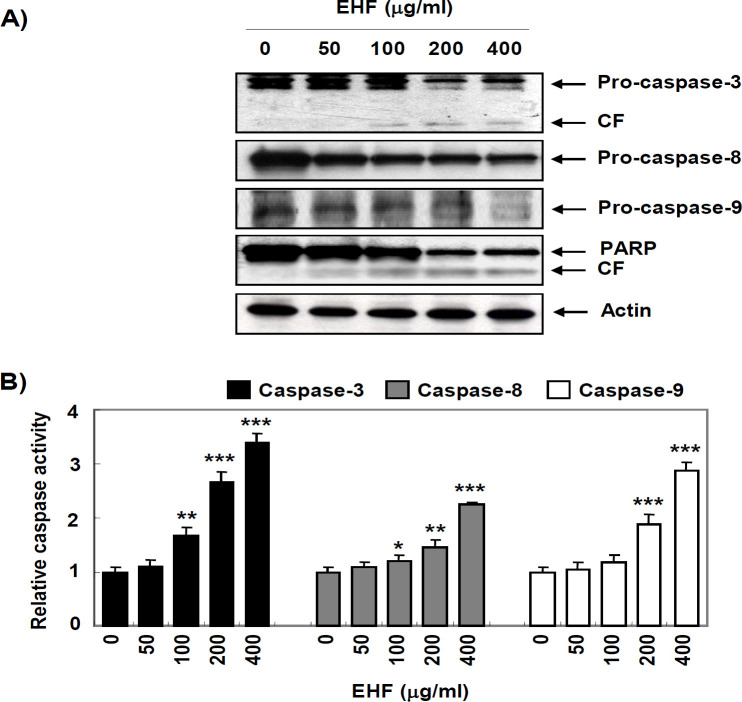
Activation of Caspases and Degradation of PARP by EHF in B16F10 Cells. B16F10 cells were treated with various concentrations of EHF for 48 h. (A) The cell lysates were prepared and equal amounts of cellular proteins were separated on SDS-polyacrylamide gels and transferred to membranes. The membranes were probed with the indicated antibodies and the proteins were visualized using an ECL detection system. The equivalent loading of proteins in each well was confirmed by actin. The results shown are representative of three independent experiments. (B) The activities of caspases were evaluated using caspases colorimetric assay kits. The data are expressed as mean ± SD (n=3). The statistical analyses were conducted using analysis of variance between groups (*P<0.05, **P<0.001 and ***P<0.0001 compared to control)

We next assessed whether EHF activated the caspase signaling pathway in B16F10 cells and found that EHF apparently reduced the expression of pro-caspase-3, -8 and -9 (Figure 2A). EHF also induced degradation of poly (ADP-ribose) polymerase (PARP), one of the representative substrate proteins of activated caspase-3 (Figure 2A). Consistent with the Western blotting results, the activity of caspases-3, -8 and -9 were significantly increased by EHF in a concentration-dependent manner (Figure 2B). 

*Mitochondrial dysfunction by EHF in B16F10 cells*

**Figure 3:**
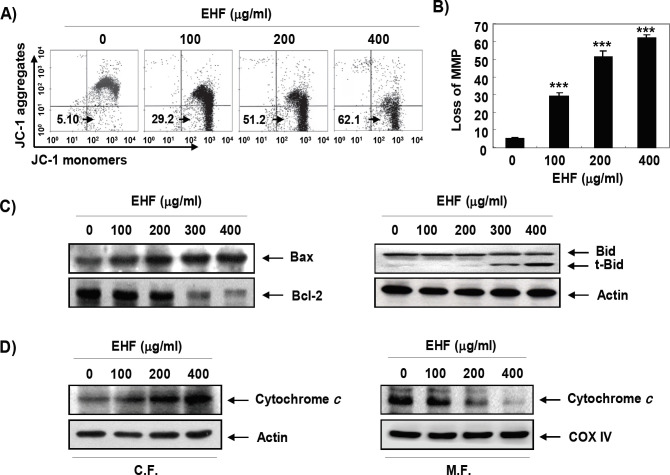
Induction of Mitochondrial Dysfunction and Cytosolic Release of Cytochrome c by EHF in B16F10 Cells. (A) After 48 h treatment with varying concentrations of EHF, the cells were stained with JC-1 dye and were then analyzed on a flow cytometer in order to evaluate the changes in MMP. (B) Data are expressed as mean ± SD (n=3). Statistical analyses were conducted using analysis of variance between groups (***P<0.0001 compared to control). (C) The cell lysates were prepared and the expression of Bcl-2 family proteins (Bcl-2, Bax and Bid) was evaluated by Western blot analysis with whole cell lysates. Equal protein loading was confirmed by an analysis of actin. (D) Cytosolic and mitochondrial proteins were prepared and analyzed for cytochrome c expression by Western blot analysis. Equal protein loading was confirmed by analysis of actin and cytochrome oxidase subunit VI (COX VI) in each protein extract. The results shown are representative of three independent experiments

We further evaluated whether mitochondrial dysfunction was involved in the induction of EHF-induced apoptosis using JC-1 dye, an indicator of MMP. As can be seen from the results by JC-1 staining, the MMP-dependent formation of JC-1 aggregates in mitochondria was maintained at a relatively high rate in cells not treated with EHF (Figures 3A and B). However, JC-1 aggregates were significantly reduced after treatment with EHF in a concentration-dependent manner, indicating a significant depletion of MMP after EHF treatment. As indicated in Figure 3C, we also found that EHF decreased the expression of anti-apoptotic Bcl-2 and increased the expression of pro-apoptotic Bax. In addition, the expression of truncated BH3 interacting-domain death agonist (tBid) was increased and the cytosolic release of cytochrome c was promoted in EHF-treated B16F10 cells (Figures 3C and D). 

*ROS-dependent mitochondrial dysfunction and apoptosis by EHF in B16F10 cells*

**Figure 4:**
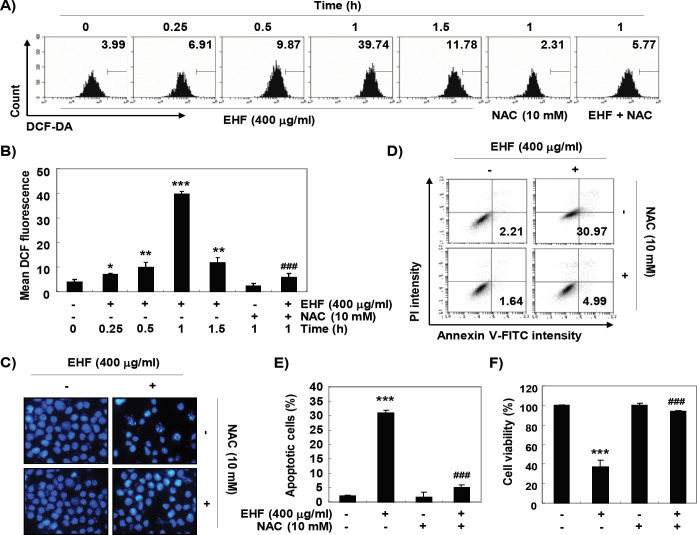
Induction of ROS-Dependent Apoptosis by EHF in B16F10 Cells. (A and B) B16F10 cells were treated with 400 μg/ml EHF for the desired times, or pretreated with or without 10 mM NAC for 1 h and then treated with 400 μg/ml EHF for 1 h. The medium was discarded and the cells were incubated at 37°C in the dark for 20 min with new culture medium containing DCF-DA. ROS generation was measured by a flow cytometer. (B) Each bar represents the mean ± SD of three independent experiments (*P<0.05, **P<0.001 and ***P<0.0001 compared to control; ###p < 0.0001 when compared to EHF-treated cells). (C-E) The cells were either treated with 400 μg/ml EHF for 48 h or pre-treated with 10 mM NAC for 1 h before EHF treatment. (C) The cells were collected, fixed, and stained with DAPI solution. The stained nuclei were pictured under a fluorescence microscope. (D) The cells were collected and stained with FITC-conjugated Annexin V and PI for flow cytometry analysis. (E) The percentage of apoptotic cells are shown as the mean ± SD (n=3). The statistical analyses were conducted using analysis of variance between groups (***P<0.0001 compared to control; ^###^*p* < 0.0001 when compared to EHF-treated cells). (F) Cell viability was measured by MTT assay. Each bar represents the mean ± SD of three independent experiments (***P<0.0001 compared to control; ^###^p < 0.0001 when compared to EHF-treated cells).

We next examined whether the production of ROS was increased by EHF and the effects of increased ROS on EHF-induced apoptosis and growth inhibition. As a result of examining the generation of ROS using DCF-DA, the accumulation of ROS was highest after 1 h of EHF treatment, and then gradually decreased thereafter (Figure 4A and B). However, cells co-treated with NAC, a potent ROS scavenger, showed significantly reduced ROS levels compared to those of EHF alone treated cells (Figure 4A and B). In addition, NAC significantly prevented EHF-induced apoptosis and reduced viability in B16F10 cells (Figure 4C-F). 

*Inactivation of PI3K/Akt signaling pathway by EHF in B16F10 cells*

**Figure 5:**
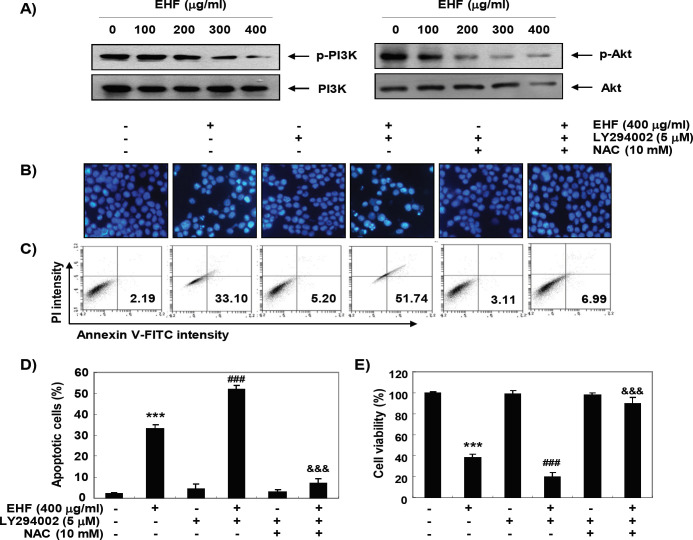
ROS-Dependent Inactivation of PI3K/Akt Signaling Pathway by EHF in B16F10 Cells. (A) After 48 h treatment with the indicated concentrations of EHF, the cell lysates were prepared and the expression of PI3K and Akt proteins was evaluated by Western blot analysis with whole cell lysates. (B-E) The cells were pre-treated with 10 μM LY294002 and/or 10 mM NAC for 1 h and then treated with 400 μg/ml EHF for further 48 h. (B) The DAPI-stained nuclei were then observed with a fluorescence microscope. The results shown are representative of three independent experiments. (C) The cells were collected and stained with FITC-conjugated Annexin V and PI for flow cytometry analysis. (E) The percentage of apoptotic cells are shown as the mean ± SD (n=3). The statistical analyses were conducted using analysis of variance between groups (***P<0.0001 compared to control; ^###^*P* < 0.0001 when compared to EHF-treated cells; ^$$$^*P* < 0.0001 when compared to EHF and LY294002-treated cells). (E) Cell viability was measured by MTT assay. Each bar represents the mean ± SD of three independent experiments (***P<0.0001 compared to control;^ ###^*P* < 0.0001 when compared to EHF-treated cells; ^$$$^*P* < 0.0001 when compared to EHF and LY294002-treated cells).

To determine the effect of EHF on the PI3K/Akt signaling pathway, we measured the phosphorylation level of PI3K protein and its downstream component Akt. As shown in Figure 5A, when cells were exposed to EHF, the expression of phosphorylated (p)-PI3K and p-Akt was gradually decreased with increasing concentration of EHF treatment, while total PI3K and Akt protein levels remained constant during EHF treatment, suggests that EHF was able to block the activation of the PI3K/Akt pathway in B16F10 cells. To further confirm the role of the PI3K/Akt pathway in EHF-mediated apoptosis, cells were co-treated with LY294002, a specific PI3K inhibitor, and EHF. The results obtained from DAPI staining and flow cytometric analysis showed that apoptosis was significantly increased in cells treated with PI3K inhibitor and EHF compared to EHF alone (Figure 5B-D). In addition, the reduction of cell viability by EHF was reduced after combination treatment with LY294002 and EHF (Figure 5E).

*ROS-dependent inactivation of PI3K/Akt signaling by EHF in B16F10 cells*

We further investigated the role of PI3K/Akt signaling pathway on ROS generation-mediated apoptosis by EHF. As shown in Figure 5B, C and D, when the production of ROS was artificially blocked, the increased apoptosis induced by the co-treatment of EHF and LY294002 as observed with the nuclear morphological changes and flow cytometric analysis was significantly protected. Consistent with these results, the reduced cell viability by co-treatment with EHF and LY 294002 was also significantly restored by blocking ROS production (Figure 5E).

## Discussion

The present study explored the anti-cancer activity of EHF, ethanol extract of H. fusiforme, in B16F10 mouse melanoma cells. EHF activated caspase-8 and -9, which are initiator caspases for activation of extrinsic and intrinsic pathways, respectively, and increased the truncation of Bid in B16F10 cells. In addition, EHF induced mitochondrial dysfunction, which was accompanied by a downregulation in the Bcl-2/Bax ratio and promotion of cytosolic release of cytochrome c. The activity of caspase-3 was also significantly increased by EHF treatment, and the cleavage of PARP was induced. 

Accumulated evidence has shown that mitochondria are considered an important target of ROS damage, and an increase in ROS levels can cause apoptosis by oxidizing mitochondrial pores (Galadari et al., 2017; Moloney and Cotter, 2018). High levels of ROS are known to modulate cancer-promoting cascades including proliferation, migration and metastasis, by leading to oxidized DNA, lipids, and proteins, thus compromising their structure and function and participating in the execution of cell death through the activation of extrinsic and/or intrinsic pathways (Galadari et al., 2017; Badrinath and Yoo, 2018). Our results showed that ROS generation was markedly increased by EHF treatment, which was greatly suppressed by the ROS scavenger, NAC. We also confirmed that EHF-induced apoptotic events were all fully blocked by quenching of ROS generation, demonstrating that EHF-induced apoptosis of B16F10 cells was clearly ROS-dependent.

Meanwhile, as is well known in previous studies, Bid, a pro-apoptotic protein belonging to the Bcl-2 family proteins, is cleaved and converted to tBid by caspase-8 activated by the initiation of the extrinsic pathway (Yin, 2006; Billen et al., 2009; Edlich, 2018). tBid in turn translocates to the mitochondria to promote the permeability of the mitochondrial outer membrane, leading to accumulation of cytochrome c in cytosol and counteracting the cytoprotective activity of Bcl-2 protein, and thereby amplifying the intrinsic pathway (Yin, 2006; Kantari and Walczak, 2011). Therefore, the current results indicate that EHF induced apoptosis in B16F10 cells by simultaneously activating the ROS-dependent extrinsic and intrinsic pathways through tBid-mediated crosstalk. 

Abnormal activation of the PI3K/Akt signaling pathway has recently been shown to be involved in the pathogenesis of multiple human tumors including melanoma (Kwong and Davies, 2013). Activated PI3K initiates activation of Akt, a downstream kinase of PI3K, which can inhibit apoptosis by protecting caspase cascade through phosphorylation of caspase-9 and promote the expression of anti-apoptotic proteins of the Bcl-2 family proteins, thereby enhancing cell survival and proliferation of cancer cells (Song et al., 2006; Manning and Cantley, 2007). Because these ultimately contribute to resistance to chemotherapy for inducing apoptosis in cancer cells, PI3K and its regulatory factors are attractive targets for cancer treatment. Therefore, we analyzed whether this signaling pathway was involved in the induction of B16F10 cell apoptosis by EHF, and found that the PI3K/Akt signaling pathway is inactivated by EHF treatment. Furthermore, LY294002, a pharmacological inhibitor of PI3K, significantly enhanced the cytotoxic effect of EHF, supposing that EHF-induced apoptosis is induced by blocking the PI3K/Akt signaling pathway. 

Recent studies have reported that several bioactive compounds generated ROS to activate apoptosis signaling in cancer cells, while ROS-dependent suppressing the activity of the PI3K/Akt signaling pathway (Mi et al., 2016; Song et al., 2017; Guo et al., 2018). These observations suggest that inducing the production of ROS in cancer cells can be used in therapeutic strategies such as induction of apoptosis through inhibition of cell survival signals such as PI3K/Akt. According to our results, the dephosphorylation of PI3K and Akt proteins by EHF was markedly attenuated in the presence of NAC and the increased apoptosis and reduced cell viability by co-treatment of EHF and LY294002 was also significantly blocked by pretreatment of NAC. However, further studies are warranted to determine the direct relationship between EHF-mediated inactivation of PI3K/Akt signaling pathway and other cellular signaling pathways, and the identification and role of intracellular organelles involved in ROS generation by EHF.

Taken together, our results lead us to suggest that the production of ROS by EHF plays a critical role in the induction of apoptosis through simultaneous initiation of both extrinsic and intrinsic pathways in B16F10 cells through inactivation of the PI3K/Akt signaling pathway. Although it is also necessary to identify the physiologically active components contained in EHF and to re-confirm their anticancer efficacy through animal experiments, EHF may be an effective and selective anti-cancer strategy for cancer based on a therapeutic natural source.
